# Ethical issues in using Twitter for population-level depression monitoring: a qualitative study

**DOI:** 10.1186/s12910-016-0105-5

**Published:** 2016-04-14

**Authors:** Jude Mikal, Samantha Hurst, Mike Conway

**Affiliations:** Minnesota Population Center, University of Minnesota, Twin Cities, 50 Willey Hall, 225 – 19th Avenue South, Minneapolis, MN 55455 USA; Department of Family Medicine & Public Health, University of California, San Diego, MTF 162E, 9500 Gilman Drive, La Jolla, CA USA; Department of Biomedical Informatics, University of Utah, Rm 2008, 421 Wakara Way, #140, Salt Lake City, UT USA

**Keywords:** Depression, Ethics, Social networking, Twitter messaging

## Abstract

**Background:**

Recently, significant research effort has focused on using Twitter (and other social media) to investigate mental health at the population-level. While there has been influential work in developing ethical guidelines for Internet discussion forum-based research in public health, there is currently limited work focused on addressing ethical problems in Twitter-based public health research, and less still that considers these issues from users’ own perspectives. In this work, we aim to investigate public attitudes towards utilizing public domain Twitter data for population-level mental health monitoring using a qualitative methodology.

**Methods:**

The study explores user perspectives in a series of five, 2-h focus group interviews. Following a semi-structured protocol, 26 Twitter users with and without a diagnosed history of depression discussed general Twitter use, along with privacy expectations, and ethical issues in using social media for health monitoring, with a particular focus on mental health monitoring. Transcripts were then transcribed, redacted, and coded using a constant comparative approach.

**Results:**

While participants expressed a wide range of opinions, there was an overall trend towards a relatively positive view of using public domain Twitter data as a resource for population level mental health monitoring, provided that results are appropriately aggregated. Results are divided into five sections: (1) a profile of respondents’ Twitter use patterns and use variability; (2) users’ privacy expectations, including expectations regarding data reach and permanence; (3) attitudes towards social media based population-level health monitoring in general, and attitudes towards mental health monitoring in particular; (4) attitudes towards individual versus population-level health monitoring; and (5) users’ own recommendations for the appropriate regulation of population-level mental health monitoring.

**Conclusions:**

Focus group data reveal a wide range of attitudes towards the use of public-domain social media “big data” in population health research, from enthusiasm, through acceptance, to opposition. Study results highlight new perspectives in the discussion of ethical use of public data, particularly with respect to consent, privacy, and oversight.

## Background

Twitter, a microblogging platform with 320 million global users in 2015, is used by 23 % of all adult internet users in the United States [1], and has emerged as an important resource for understanding attitudes and behavior at the population level, particularly in such areas as business and marketing [2, 3], election monitoring [4, 5], and gauging public opinion on important policy issues [6, 7]. In the public health domain, the potential of Twitter – and other “big data”^1^ social media sources available in machine readable format – to enhance population-level health monitoring is increasingly recognized (e.g. [8–11]), with applications such as detection and monitoring for early-stage disease outbreaks [12], influenza monitoring [13–15], public health surveillance for mass gatherings [16], recognizing temporal variability in lifestyle and health behaviors [17], and investigating public attitudes towards emerging tobacco products like electronic cigarettes and hookah [18]. More recently, significant research has focused on using Twitter (and other social media) to investigate mental health (and risk factors associated with mental health) at the population-level [19], including work on correlating suicide-related keywords with United States suicide rates [20] and automatically identifying depression symptoms [21–23]. Despite the potential of publicly available social media data, in combination with computationally efficient Natural Language Processing (NLP) techniques [24], to augment current telephone-based public health monitoring efforts (e.g., in the United States, the Behavioral Risk Factor Surveillance System [25]), significant doubt remains among regulatory authorities and research ethics committees regarding ethically appropriate uses for these new data sources. This is particularly true in the wake of Facebook’s 2014 “emotional contagion” intervention study [26], and concerns expressed regarding *Samaritans Radar*, a Twitter app designed by Samaritans UK – a suicide prevention charity – to monitor the tweets of a user’s contacts for evidence of suicidal ideation [27].

While there has been influential work in developing ethical guidelines for Internet discussion forum-based research in the health domain [28], limited work has focused on addressing ethical problems in big data Twitter-based public health research. Notable exceptions include Vayena 2014 [29], who identified three broad themes of key importance for big data public health research (“context sensitivity”, “nexus of ethics and methodology”, and “legitimacy requirements”), and Conway 2014 [30], who constructed a taxonomy of ethical concepts relevant in using Twitter for public health research and surveillance from the research literature (including concepts, “privacy”, “informed consent”, and, “regulation”). While these studies provide insights into the ethical issues surrounding public health monitoring using new, publicly available data sources, they primarily address researcher beliefs, current regulation, and bioethical theorizing.

Research that has addressed user attitudes towards access to social media data more generally [31, 32], has generated equivocal findings. Where Beninger [31] finds conditional acceptance of using social media data for research purposes, Evans [32] finds that nearly 60 % of users do not support the use of social media data for research. These inconsistent findings may result from the fact that both studies treat social media data and resulting research very generally. As such, the studies do not report user attitudes towards NLP and big data, specifically, nor do they clearly distinguish between user attitudes towards “broadcast” social media services like Twitter and closed platforms like Facebook, where users are able to use more fine-grained privacy controls. Very little is known about the attitudes of *individual Twitter users* regarding the use of their data for population health monitoring, specifically as it might relate to potentially stigmatized mental health conditions.

In this study, we analyze the attitudes and ethical beliefs of Twitter users towards the use of their public domain data for population-level health monitoring, particularly focusing on mental health issues. We conducted five, in-depth focus group interviews with Twitter users with and without a history of diagnosed depression – 26 participants in total – to investigate bioethical questions arising from emerging technologies. Our semi-structured interview was centered around five themes: (1) Twitter use patterns, (2) privacy expectations, (3) attitudes towards population level mental health monitoring, (4) differences between individual and population level health monitoring, and (5) users’ own recommendations regarding regulation (or developing ethical guidelines for) population-level health monitoring using social media. While the study aims to provide insights into participants’ understanding and attitudes towards aggregate mental and public health monitoring, a further key goal is the generation of hypotheses and research questions to guide future investigation.

## Methods

### Preliminary work

The goal of this study is to investigate public opinions on the use of Twitter data for population health monitoring generally, and population mental health monitoring in particular. In order to refine the research question, evaluate the effectiveness of different recruitment strategies, and assess participant responses to potentially sensitive questions, we conducted a pilot study consisting of three online Skype interviews. Participants were recruited via online forums (two participants identified as having a history of depression, one did not) and verbally consented. User responses were employed to frame the semi-structured protocol for the main study. Furthermore, our pilot work indicated that recruiting from local community Internet sites (e.g. Craigslist, Reddit) was likely to be a more fruitful strategy than recruiting from anonymous online mental health forums, where the response rate was very low.

### Research design

We used a focus group interview strategy for the main part of the study in order to encourage the spontaneous generation of ideas through group dialog and interchange. Focus groups are considered an ideal avenue for the exploration and clarification of new ideas. They also serve to empower participants as an integral part of research and analysis, emphasizing interactions between participants, and de-emphasizing the role of the interviewer [33].

### Recruitment and sampling

Given the focus on mental health monitoring, we organized Twitter users into two groups: users *with* a diagnosed history of depression, and users *without* such a diagnosis. We advertised for Twitter users with and without a history of professionally diagnosed (as opposed to self-diagnosed) depression. Note that any diagnosis was self-reported. Participants for both groups were recruited through list serves, Internet discussion boards, and flyers. The general announcement included information about the study, inclusion criteria, and information on remuneration, along with researchers’ contact information. We did not require a minimum threshold Twitter use – e.g. a minimum number of tweets – for eligibility. Our most successful avenue for recruitment was Reddit.com/[city name], where we posted our flyer three times. It is important to note as a limitation that Reddit users tend to skew young and male; 6 % of online adults report using Reddit, with men twice as likely as women to be users [34]. The study was conducted in the western United States.

### Focus groups

Five focus groups took place between March and April 2015. The groups were conducted face-to-face, were audio recorded, and lasted 2 h each. Two control groups with five non-depressed participants were conducted first (*N* = 5, *N* = 5), followed by three focus groups with participants with a history of diagnosed depression (*N* = 4, *N* = 4, and *N* = 8, respectively). Verbal consent was gained from each participant. The focus group protocol consisted of questions grouped around five main themes:

1. Twitter use
2. Privacy expectations
3. Population mental health monitoring
4. Individual versus aggregate mental health monitoring
5. Participant views on regulating social media mining

Following Hennink [35] to encourage participation, focus groups began with introductions in which each participant stated their name (or preferred pseudonym), age, occupation, and general Twitter use patterns. Topics were introduced in a series of “grand tour”, or open-ended questions to enable participants to determine the direction of the discussion. We used probing questions (mini-tour, example, experience, and language) to both encourage conversation, and to understand the participants’ experiences [36, 37]. A complete protocol, including both grand tour and probing questions can be found in Appendix A.

### Data analysis

Following the model of Pope et al. [38], focus groups were subjected to interim analysis, and the protocol was adapted to explore and triangulate findings from earlier interviews. Authors JM and MC met to review audio recordings, and discuss emergent themes. Once all focus group interviews were completed, the audio recordings were sent to a professional transcription service to be transcribed. Transcriptions yielded 282 single-spaced pages of text. Authors JM and MC independently conducted a constant comparative analyses – using an inductive approach to allow themes to emerge from the data itself, guided by our research foci [39]. The authors then met to review and decide upon salient themes. The authors, guided by the initial research question, decided on five descriptive content domains. Coding was then validated by author SH, and subsequently written up for publication.

### Participant characteristics

In accordance with IRB requirements, would-be participants were provided with researchers’ contact information and asked to contact researchers directly if they were interested in participating. More than 35 people responded to our recruitment advertisements. Of those, 26 were willing, eligible and verbally consented to sit for focus group interviews. Our participants were predominantly male (17 M/8 F). The population skewed young with an average age of 26.9 years (stdev = 8.8 years), and an age range between 19 and 54 years old. Of our total 26 respondents, 16 reported a diagnosed history of depression and ten reported no depression history. Participants were from various professions, which were divided into six categories: computer/technology (five respondents), office/administrative (eight respondents), education (one respondent), students (seven respondents), specialized services (four respondents), and stay-at-home parent (one respondent). Twitter use ranged from passive/content receiving to active/content generating [40]. Table 1 presents participant characteristics.

**Table 1:** Participant characteristics and Twitter use patterns

Group	Age	Sex	Twitter use
FG1: Control	27	M	Professional, social interaction, venting
FG1: Control	22	M	Professional
FG1: Control	26	F	Following, social interaction
FG1: Control	19	M	Following
FG1: Control	22	F	Social interaction, following
FG2: Control	29	M	Following
FG2: Control	21	F	Social interaction
FG2: Control	21	F	Professional
FG2: Control	40	F	Following, social interaction
FG2: Control	24	M	Professional, social interaction
FG3: Depression	29	M	Professional, following
FG3: Depression	20	M	Social interaction
FG3: Depression	29	M	Social interaction, following
FG3: Depression	54	M	Social interaction, following
FG4: Depression	42	M	Professional
FG4: Depression	21	F	Social interaction
FG4: Depression	23	M	Following
FG4: Depression	33	M	Social interactions, professional
FG5: Depression	20	F	Following
FG5: Depression	18	M	Following, professional, social interaction
FG5: Depression	30	M	Following, venting
FG5: Depression	22	M	Following, social interactions
FG5: Depression	22	M	Professional, following
FG5: Depression	21	F	Professional
FG5: Depression	24	F	Social interactions
FG5: Depression	31	M	Professional, social interactions

## Results

Regarding the use of public social media data for population health monitoring, our analysis revealed a range of opinions, from enthusiasm, through acceptance to opposition. Users accepted a sense of personal responsibility for what they posted, and viewed use of the data generated as a price for participation on Twitter. In this section we examine the responses from participants to our semi-structured interview.

### Twitter use – different ways that participants use Twitter

Patterns of use: Four broad patterns of use emerged from the data. Some users reported engaging in *professional promotion*, either for their own independent business ventures, or as the social media representative for a larger business. Several users reported using Twitter for *social engagement*, generally interacting with peers, or other Twitter users based on common interests, sharing thoughts, or participation in particular events. A third type of Twitter use was *venting*. While fewer people reported venting as a separate category, respondents reported using Twitter to interact with businesses as empowered consumers by raising public awareness of poor quality goods/services. Finally, respondents reported using Twitter to *follow* content generators, staying up-to-date with news, events, content, and promotions. *Professional promotion, social engagement, and venting* are classified as *active/content generating* uses, while *following* is classified as *passive/content receiving*.

### Privacy expectations – do participants have different understanding of their level of privacy on Twitter?

“You are the product”: Many users disavow the expectation of privacy. Twitter is a public forum, they report, and as such there is no assumption of privacy. According to one participant, the fact that Twitter is free is important,

*I don’t pay to use Twitter. I sort of signed up with the expectations that it’s a free site and you just kind of throw things out publicly, [so] I don’t really have an expectation that anything that I post is going to remain private* [Control group, 29, M].

Another respondent in focus group three echoed a similar sentiment with a more negative tone. In response to another participant’s comment that Twitter needs to turn a profit somehow, Phillip says,

*Exactly, like that’s what their product is. Their product is you. Because it’s free, you are the product* [Depression group, 29, M].

Despite this commonly held understanding, our focus group data revealed that some privacy is expected. In fact, while some users state outright that they assume no privacy, the expectation of privacy may still remain intact given users’ (1) failure to understand data permanence, (2) failure to understand data reach, and (3) failure to understand the big data computational tools that can be used to analyze posts (discussed below).

Perception that data is ephemeral: One common misconception about Twitter data was that the data was ephemeral. The Twitter users interviewed were under the impression that accounts could be manicured, or that information generated before a certain date could not be retrieved (i.e. the Twitter user interface, and the computational and data infrastructure that supports that interface, were conflated by the users). In response to whether there was any potentially “incriminating” information on her Twitter account, one participant said,

*I would say definitely. <chuckles> Maybe it’s because I’m young, so I started into social media when I was younger, like really young. So every once in a while, I’ll go through [and delete]* [Control group, 21, F]*.*

Deleting posts suggests a possible misconception regarding what data remains after deletion.^2^ Another participant also reflects similar ideas regarding the permanence of Twitter data saying,

*I would say most of the time I’m not afraid to rock the boat. But I mean, Twitter won’t let you scroll back that far, so I’m not super concerned* [Depression group, 20, M]*.*

A further participant was not under the impression that Twitter data could not be accessed, but felt as though the amount of data and text he generated made posts more difficult to find. In reference to a *sub-tw*eet – i.e. a critical tweet that refers to an individual without explicitly naming them – made in response to a relationship breakup, he says,

*I had to scroll through probably 200 to 300 tweets until I could find that sub-tweet. And I think especially in the last year as I’ve been getting more followers, I’ve been more aware of what I’ve been tweeting* [Depression group, 22, M].

These statements suggest that, despite users’ understanding that Twitter is public, they may not be aware of the extent to which Twitter data is permanent, and available to anyone via the (free) Twitter Application Programming Interface or via data reselling services^3^.

Data Reach: Another area that pointed to some misconception with regards to privacy was Twitter users’ conceptualizations of data reach. In response to another users’ privacy concerns, one participant retorted,

*Are you naïve enough to think that your public tweet is going to be seen by like a million people? I mean sure, it’s public. Anyone could go and find it, or search for it, or whatever. I mean, but it’s not like Beyoncé tweeting is the same as me tweeting* [Depression group, 54, M].

Nevertheless, many users demonstrated a lack of understanding with regards to the potential reach of their own data. Users in both focus group two and focus group five justified their lack of care with Twitter data by saying that they only had a handful of followers. However, one participant describes the problem with tweeting to a select group of followers,

*You don’t really think about the far-reaching amount of people that can actually use what you say* [Depression group, 29, M].

And several users discuss humorous tweets they made that were favorited by friends, and thus reached individuals they may not have chosen to share jokes with otherwise.

The Choice to Personalize Privacy Settings: Many users felt as though methods were available to them to limit their online presence. For some this included setting your account to “private”, for others it was deleting accounts and disappearing from social media altogether. Failure to personalize your online presence and online settings constituted an implied consent to having your data collected and analyzed. According to one participant,

*You’re voluntarily using Twitter. So it goes back to that whole: the Internet’s public domain. If you want to have your data combed through, then please continue to post things on the Internet* [Control group, 21, F].

The notion that interacting online, and in a public forum, implied giving consent to have data amassed and analyzed was echoed by focus groups in each of the five interviews. However, some participants’ views were more tempered. For example, some participants felt as though it was the choice of website that implied agreement to be used in datasets (i.e. Twitter is presumed to be a public platform by default, in contrast to Facebook which has explicit privacy controls). According to one respondent,

*It all comes down to the fact that we know that we’re using Twitter and it’s public. I think I might honestly feel differently about that if it were Facebook, because I do feel like there is some degree of privacy in Facebook* [Control group, 21, F].

For this participant, the auspices under which information is shared and the knowledge that data is public, permits users to exercise control, and to manage and edit self-disclosure.

Personal Responsibility: For many, Twitter use came down to a question of personal responsibility. For these respondents, Twitter presence, and online presence in general is a matter of personal choice. Two participants in separate focus groups referred to social networking, and the resultant data as the “Wild West”, existing outside of formal laws and regulation. As a result, many participants felt as though users had a personal responsibility to ensure their own comfort with the data that was generated. According to one participant,

*I think our generation is gravitating towards [the idea that] privacy is not to be expected anymore. You have to create it yourself. You have to enable it yourself, because it just doesn’t exist anymore* [Control group, 27, M].

Even the most privacy-conscious users acknowledged that lingering evidence of their online activity was a matter of personal choice. According another participant,

*I just acknowledged to myself a long time ago that whatever I put on the Internet - whatever I put into my search engine, anything that I click on – is not private*. [Depression group, 21, F].

Implied in these statements was not the notion that no oversight or regulation was necessary; only that in an environment devoid of such regulation, users needed to be careful with the evidence they left behind online.

### Population health monitoring (particularly depression) – participants’ views on using Twitter to monitor disease at the population level

Population level data: Respondents expressed optimism regarding the use of Twitter data for public health at the aggregate level. While some users expressed concerns regarding privacy, others felt as though service to the greater social good was more important than individual privacy concerns. When asked to discuss the issue of Twitter use for aggregate public health monitoring, one participant states,

*I kind of think it’s cool when it’s stuff that’s like the flu, because then that’s how they know to get the vaccines to a place* [Depression group, 24, F].

When in the service of public health, other respondents were also willing to put aside privacy concerns. One participant articulates a particularly open viewpoint that was echoed by other members of that focus group interview:

*I can’t be in a position to know all the possible things that someone could come up with, all the beneficial things, all the harmful things. I think [it represents one-percent of the issues], the whole array of things that are possible shouldn’t be stopped because we’re so overly worried about [privacy]* [Depression group, 54, M].

While this attitude is somewhat more strongly worded than the attitudes of other participants, users generally took a utilitarian stance towards open access, provided that studies were in service to the greater good:

*It’s like fluoride in the water to me. They put fluoride in our water. We don’t really have a choice if we want to drink water, we’re going to get fluoride. But the benefits outweigh the risk* [Control group, 26, F].

Privacy concerns for these participants were rendered less significant by the potential of Twitter to provide current, accurate information in service of the greater public good.

When asked about the use of Twitter data in public health monitoring, most members echoed the sentiments of the two participants who replied, “I have no problem with that.” Yet, even at the aggregate level, two users from separate focus groups characterized the use of Twitter data to monitor depression as “creepy”. One participant, who is otherwise in support of the use of publicly available data social media data for population health applications, conveys a sense of unease,

*You’re screaming into the void, and someone is listening. It’s a little bit creepy, but it’s taking the words from your own mouth* [Control group, 21, F].

When probing questions were used to unpack the concept of “creepy”, user responses indicated a difficulty in distinguishing between aggregated and disaggregated data, citing concerns about privacy, or how being identified as having a high likelihood of depression might impact an individual. According to one participant,

*The fact that if it was an algorithm, and they were looking like, ‘Hey, we think you’re feeling low right now.’ I feel like it might make me feel even more low* [Depression group, 24, F].

Other users commented on the potential for words to be taken out of context, compromising confidentiality, or the stigma faced by individuals suffering from mental health issues. However, these concerns generally resulted from the ability to target particular individuals, rather than aggregate level mental health monitoring.

Yet even for the most enthusiastic supporters of public health monitoring, permissions were not without qualification. While several participants were comfortable providing complete access to their Twitter data, many stipulated that permission could only be implied where it pertained to aggregated, anonymized data:

*I think I would be more comfortable being identified just in the group. So having somebody not be able to be like, ‘Oh, this specific Twitter name has the flu virus.’ Instead, ‘Just this many people have it.’ And there’s not like specific data that could be identified out of that group* [Depression group, 24, F].

Another participant expresses a similar viewpoint in response to a question regarding mental health monitoring, in particular,

*I’m OK as long as we can, you know, figure out ways to keep the data anonymous and completely, highly aggregated* [Depression group, 42, M].

This general aggregated monitoring of public health outcomes using Twitter, including aggregate population-level rates for depression, met with qualified support from participants. The concerns of participants who expressed a continued reluctance to support the use of even aggregated data could be categorized under two themes: accuracy and unintended consequences. These issues will be discussed in more detail, below.

Accuracy: While many users reported that their own experiences with depression could be observed from their past social networking behavior, a major theme that emerged from the focus group findings was that Twitter data may not be an accurate proxy for underlying mood – and may produce aggregate depression rates that are unreliable. Users were principally concerned that the ways in which depression was likely to manifest may not be captured by simple keyword matching algorithms. Users were also concerned that the ways in which they used Twitter, and the content they generated, would not produce reliable data.

Each of the three focus groups with individuals who had been diagnosed with depression was asked, “Do you feel like your depression, or your experiences with depression would be evident from your online interactions?” One participant responds that his social networking behavior would be indicative of his mental state. He tells the story of a bout of depression during his senior year of high school (i.e. around 18 years of age) saying,

*During my senior year, I would just tweet just because I wanted my friends to see it and to know that I didn’t feel good, or that I was upset or mad at someone. I think it would be very obvious, actually* [Depression group, 20, M].

According to another participant, this may be true of people in general. He suggests that looking at students’ social networking data during finals might provide some insights into the lived experiences of students,

*If you look at a student’s Facebook or Twitter, especially during finals time, you see how stressed people are. You see people aren’t sleeping. They aren’t eating. All they’re doing is studying, and their moods are getting worse and worse on social media* [Depression group, 31, M].

Despite this feeling, some participants remained skeptical. “You can’t even get targeted advertising right,” quipped one participant, “what makes you think public health accuracy is going to be any better?”

Nevertheless, by looking at other ways in which users manifest their depression, public health monitoring could be improved. Consistent with the known relationship between depression and social isolation [41], several participants were concerned that automatic monitoring may miss cues such as decreased activity:

*It’s just the opposite for me. If I’m feeling down or anything, I just kind of retreat back. There’d be a huge gap there* [Depression group, 29, M].

Also commonly cited as an accuracy concern was the issue of falsehood – which was likely to take many forms. Users’ concerns related less to lying on social media. Rather, they discussed issues related to multiple accounts, false positivity, and what they felt was the appropriate content for social media. According to one participant,

*I’ve never once posted anything negative. So if you took that data, it would not be accurate, because of course I have had bad days or sad days* [Control group, 40, F].

### Diagnostic versus aggregate health monitoring – differences between population level monitoring and individual diagnosis

Concerns: The potential for disaggregation of data to identify individuals who may be suffering from depression was met with mixed response. Users were concerned that the tools used to predict aggregate rates of depression at the population level could also be used to pinpoint individuals suffering from depression. This could lead to identification and further stigmatization. According to one participant,

*Once you’ve got the taint of depression – mental illness at all in our society, it’s an uphill battle. Even now, people in my family are like, ‘Oh, you sound cranky. Have you taken your meds?* [Depression group, 33, M].

Nevertheless, several respondents felt as though pinpointing individuals could help them access much-needed mental health services by paying attention to cues that friends may ignore. The following encounter took place during focus group two:

*[Control group, 21, F]: People say things on the Internet they would never say in real life.*

*[Control group, 21, F]: That’s very true.*

*[Control group, 29, M]: I was just going to say, this probably makes me a bad person, but whenever I get the vague like “My life is terrible” Facebook posts, I just unfollow that person.*

*[Control group, 21, F]: Seriously, they just want the attention.*

*[Control group, 21, F]: I just wish there was an eye-roll button.*

Respondents are suspicious of potential indicators of depression that appear on social media, so may simply ignore them, or unfollow the person. Given that computational methods do not ignore or unfollow, they may be particularly useful in identifying and responding to indicators of danger.

On a related topic, users expressed support for the use of social media based automated mental health technologies to augment treatment in the context of traditional mental health care (e.g. a psychiatrist, with explicit patient consent, using automated tools to monitor a patient’s mood between appointments). The idea emerged from focus group two, and was presented in the three subsequent focus groups where it met with largely positive response. When the idea was presented to members of focus group three, one participant replied,

*I’m all for that. I know when I’ve gone to therapists or my doctor or whatever, I’m not the best at reporting how I’ve been doing when I’m actually at my appointment. Especially when I go see them for the first time. That would be fantastic to have something else to either support what I think, just because I’m not reliable about accurately assessing how I’m doing* [Depression group, 29, M].

Similarly, focus group members appreciated being able to accurately assess the duration of moods. One participant suggested that responding to his therapist’s questions may become easier with the help of social media history,

[Depression group, 29, M]*: I think that sounds great! Especially, I think one common question is like, how long have you felt this way? I don’t know. I don’t know.*

[Depression group, 20, M]: *Right, exactly. Forever.*

[Depression group, 29, M]*: But if you could look at Twitter and just immediately a graph that shows mood swings over time. Absolutely!*

While users emphasized that individual consent would be required, many felt that automated social media tracking could allow a wider window of observation for the mental health practitioner, and could provide some objective evidence of mood swings, and duration which would be invaluable for predicting, diagnosing, and treating depression.

### Participant views on regulating Twitter mining – participant views safeguarding privacy

Safeguarding privacy: Respondents differed in their views on the extent to which Twitter monitoring should be regulated. While some participants felt government oversight would help to ensure the ethical use of public data, others suggested that governmental oversight could lead to Orwellian monitoring. Nevertheless, even those who expressed concern with respect to governmental monitoring could not agree on the appropriate role of government. For some, it was government access to public health data that laid the foundation for abusive governmental monitoring. According to one participant,

*For me it’s like, researchers – free access. I don’t care if they have all of it. Advertisers, they should have to pay for the access. And the government should have absolutely no access [Control group, 26, F].*

While some participants felt as though government oversight was necessary to protect the rights of users, others felt as though oversight was unnecessary, or should come from the social networking sites, themselves. However, consistent themes emerged from the focus groups regarding ethical access and use of social networking data. First, users felt as though the collection, access, and use of social networking data should be transparent. Respondents did not feel as though simple blanket language in the “terms and conditions” constituted transparency. Such language was confusing and buried in what one participant terms, “a wall of text that no one ever reads”. Knowledge that using Twitter (or other social media sites), constitutes consent to have your data collected, analyzed, and commoditized should be made plain when creating a Twitter account.

## Discussion

We conducted five in-depth focus groups with Twitter users to investigate ethical issues in the use of social media big data for population health monitoring from the users’ perspective. Overall, respondents understood that Twitter data was publicly available by default, and that the responsibility to ensure the protection of data was in the hands of the user through tools like privacy settings, self-censorship, or simply opting out of social media. Given the availability of such tools, many respondents felt as though a failure to protect online data constituted consent to have that data systematized and analyzed. Nevertheless, ethical concerns remained. In this section, we review the ethical issues surrounding implied consent, and discuss users’ own recommendations for ethical use of publicly accessible social media data for population health research.

### Principal findings

In general, respondents were not opposed to the use of publicly available data for health monitoring activities, with the stipulation that the data be anonymous and aggregated to protect the identity of the people represented. Attitudes towards aggregated health monitoring ranged from enthusiasm on the part of some participants, to acceptance on the part of others who reported that implied consent was simply the price of participation in “broadcast” social networks like Twitter.

Despite users’ overall acceptance of implied consent, significant ethical issues emerged from the focus groups that warrant further consideration. Specifically, while some users understood the reach and permanence of their “digital footprint” [42, 43] other respondents expressed misconceptions regarding the degree to which a digital footprint could be managed after the fact. Deleting posts, or the inability to scroll back through more than 3200 tweets – does not mean that data has been removed. Similarly, a limited number of followers, or the vast amount of data generated by other Twitter users does not imply anonymity. Users reported uncomfortable situations where tweets reach beyond their intended audience, and were often unaware of computational tools that can sift through tweet content for specific keywords or patterns. While these represent exciting technological developments from the vantage point of health researchers, misunderstandings regarding the reach and permanence of data raise important ethical considerations.

While most users understood the public nature of the data they generate online, and many felt as though protecting that data was a matter of personal responsibility, it is interesting to consider who may be less likely to protect their data and how that may impact potential study populations. While evidence has emerged to suggest that Internet penetration rates are equal across demographic groups, recent research points to a lingering "Digital Divide" that manifests in unequal access [44] and patterns of use [45]. Otherwise stated, some Twitter users have grown up in homes without Internet access, without mobile devices, or with limited Internet literacy skills. Others have grown up using the Internet, but with very limited understanding of the underlying technology. These groups may be more likely to leave behind the type of digital footprint picked up by researchers – and in failing to protect their data, may imply consent to have their data used for research studies. Further, according to Hargittai (2010) [46], Internet literacy is still divided on the basis of gender, ethnicity, and parental education – indicating that women, certain cultural and ethnic minorities, and those with low parental education may be more likely to unknowingly imply consent, and that aggregated data may oversample from those populations.

Further, respondents expressed concerns regarding both the raw data, as well as the aggregate numbers it generated. According to respondents, raw data could be compromised by user behavior such as self-censorship, or by the common phenomenon of maintaining multiple accounts, including role-playing, business accounts, joke accounts, and others. With respect to depression in particular, users expressed concern that using keywords such as “depression” and “sadness”, could miss other textual and non-textual indicators that someone might be depressed. Several users reported that in times of distress, they were more likely to either express positivity, or to withdraw from social media altogether.

### Limitations

Our study has three main limitations. First, the results presented here are qualitative in nature, gathered and synthesized from in-depth focus group interviews. Our sample size is small, and thus results are not generalizable. These results should be used in conjunction with other work on big data and health monitoring to provide insights into ethical issues from the end-users’ perspectives. The work was conducted in an urban region of the western United States, characterized by a large religious, conservative population. While our study population was diverse, and included both religious and non-religious participants, focus group participants may be more likely to have grown up in traditional families, or in certain religious communities, and this may have impacted the perspectives they provided. Finally, participants were recruited primarily through a community-based Reddit page. This means that we may have oversampled from Twitter users with multiple social network accounts. Further, there is some risk that we may have missed Twitter users who do not use Reddit or other social media.

## Conclusions

Previous research has created ethical typologies for the use of publicly available digital data based on theory [29], and research literature [30] with some important overlap. Specifically, studies have focused on issues of privacy expectations, data regulation, and the trade-off between individual rights and the public good. Vayena et al. [29] further addresses the issue of justice and avenues for redressing the (potential) harm caused by the creation, systematization, and analysis of big data. This study complements these existing typologies, and provides additional insights into privacy expectations, data regulation, and public/private interests from the vantage of the users themselves.

### Ethical approval

The preliminary work described was approved by the University of California San Diego Institutional Review Board (#131454). The main study was approved by the University of Utah Institutional Review Board (#00077913).

### Data sharing

Given the sensitive and detailed nature of the experiences shared, and in accordance with IRB requirements, we have elected not to make participants’ stories publicly available.

## Appendix

### Semi-structured focus group protocol – including probing questions

A. Introductions
  - Name (or pseudonym)
  - Age
  - Occupation
B. General Twitter use
  - How often do you use Twitter?
  - What are the things that you are likely to post to Twitter?
  - What might you refrain from posting to Twitter? Are there things that are too personal, or things that you worry might get you into trouble?
C. Privacy expectations
  - Do you know who is able to view/monitor your Twitter use?
  - What if I were to tell you that advertisers use Twitter to collect data on your browsing history so that they can target ads to you, personally? How does that sit with you? Is that a good thing or a bad thing?
D. Privacy expectation and health
  - What if I were to tell you that researchers and health departments can use Twitter data to predict public health epidemics like flu outbreaks? How does that make you feel?.
  - What about predicting things like depression, or health behaviors like smoking? [Diet? Drug use?] Is that information more private? Would you alter your posting behavior if you knew that type of information was being monitored?
  - New programs are being developed to predict things like depression. Does this fall into the same category as advertisements, or the flu? Or is this different?
E. Recommendations
  - How should Twitter data be used? What laws or rules would you recommend?
  - Does it make a different who is doing the monitoring (health department v. private sector v. research)?
  - Is it better to use Twitter data to predict overall rates of depression at the individual, city or state level?
F. Other questions:
  - Self-presentation: Are you the same person on Twitter as you are in real life?
  - Researchers have said that using Twitter data poses no ethical dilemma because users understand that there is no privacy assumed in tweets. How do you react to this?
  - Discussion point: differences in aggregated v. diagnostic
  - Discussion point: Facebook (other social networks) v. Twitter content

## Footnotes

1 “Big data” lacks any formally agreed definition. However, one common formulation characterizes the distinguishing features of big data – compared to more traditional data – as *Velocity*, *Volume*, and *Variety* (the “three Vs”) [47].

2 Note that Twitter’s terms of service stipulate that “all reasonable efforts” should be taken to “delete content that Twitter reports as deleted or expired” (https://dev.twitter.com/overview/terms/agreement-and-policy).

3 As of September 2015, Twitter data is sold commercially through Gnip (https://gnip.com/sources/twitter)
